# Synthetic Lignan Secoisolariciresinol Diglucoside (LGM2605) Reduces Asbestos-Induced Cytotoxicity in an Nrf2-Dependent and -Independent Manner

**DOI:** 10.3390/antiox7030038

**Published:** 2018-03-02

**Authors:** Ralph A. Pietrofesa, Shampa Chatterjee, Kyewon Park, Evguenia Arguiri, Steven M. Albelda, Melpo Christofidou-Solomidou

**Affiliations:** 1 Division of Pulmonary, Allergy, and Critical Care, Department of Medicine, University of Pennsylvania Perelman School of Medicine, 3450 Hamilton Walk, Stemmler Hall, Office Suite 227, Philadelphia, PA 19104, USA; ralphp@pennmedicine.upenn.edu (R.A.P.); kyewpark@pennmedicine.upenn.edu (K.P.); evguenia@pennmedicine.upenn.edu (E.A.); albelda@pennmedicine.upenn.edu (S.M.A.); 2 Department of Physiology, University of Pennsylvania Perelman School of Medicine, Philadelphia, PA 19104, USA; shampac@pennmedicine.upenn.edu

**Keywords:** antioxidant, asbestos, inflammation, LGM2605, lignan, macrophage, mesothelioma, oxidative stress, phase II enzymes, reactive oxygen species, secoisolariciresinol diglucoside

## Abstract

Asbestos exposure triggers inflammatory processes associated with oxidative stress and tissue damage linked to malignancy. LGM2605 is the synthetic lignan secoisolariciresinol diglucoside (SDG) with free radical scavenging, antioxidant, and anti-inflammatory properties in diverse inflammatory cell and mouse models, including exposure to asbestos fibers. Nuclear factor-E2 related factor 2 (Nrf2) activation and boosting of endogenous tissue defenses were associated with the protective action of LGM2605 from asbestos-induced cellular damage. To elucidate the role of Nrf2 induction by LGM2605 in protection from asbestos-induced cellular damage, we evaluated LGM2605 in asbestos-exposed macrophages from wild-type (WT) and Nrf2 disrupted (Nrf2^−^/^−^) mice. Cells were pretreated with LGM2605 (50 µM and 100 µM) and exposed to asbestos fibers (20 µg/cm^2^) and evaluated 8 h and 24 h later for inflammasome activation, secreted cytokine levels (interleukin-1β (IL-1β), interleukin-18 (IL-18), interleukin-6 (IL-6), and tumor necrosis factor alpha (TNFα)), cytotoxicity and cell death, nitrosative stress, and Nrf2-regulated enzyme levels. Asbestos exposure induced robust oxidative and nitrosative stress, cell death and cytotoxicity, which were equally mitigated by LGM2605. Inflammasome activation was significantly attenuated in Nrf2^−/−^ macrophages compared to WT, and the protective action of LGM2605 was seen only in WT cells. In conclusion, in a cell model of asbestos-induced toxicity, LGM2605 acts via protective mechanisms that may not involve Nrf2 activation.

## 1. Introduction

Asbestos, a naturally occurring fibrous mineral, predominantly used in construction and shipbuilding, has been associated with neoplastic diseases, such as malignant mesothelioma (MM) and lung cancer [1,2]. MM has a long latency period that could extend from 30 to 50 years and arises from the mesothelial cells of the pleura and peritoneum with a median survival of about 1 year [3,4,5]. There are currently no curative therapies other than surgery at early stages [3]. Although asbestos mining and use is restricted in most western countries, it is unfortunately a persistent global issue with continued occupational and environmental exposures of individuals.

Many studies have been dedicated to the elucidation of the mechanism of how such exposures lead to malignant transformation of the mesothelial cells and, indeed, multiple mechanisms have been implicated. These are chronic inflammation [6] and generation of reactive oxygen species (ROS) predominantly by activated macrophages which in turn induces signaling pathways resulting in activation of nuclear factor-κB (NF-κB) [7] and the Nod-like receptor family pyrin domain containing 3 (NLRP3) inflammasome [8]. Malignant mesothelioma tumor samples are associated with chronic inflammation, including macrophage infiltration and inflammatory cytokine production [9]. In addition to inflammatory cytokine secretion, the activated macrophages contribute to tumorigenesis by forming harmful ROS and reactive nitrogen species (RNS) that can, in turn, further induce DNA damage and lead to potential genomic instability by increasing tissue proliferation, and inducing tissue remodeling and angiogenesis-promoting factors, as well as agents that facilitate extravasation of tumor cells from the microenvironment [10].

Oxidative stress has been closely associated with carcinogenesis; therefore, antioxidant strategies have been the focus of multiple studies over the past several decades. In a recent review by Chikara and coworkers, the usefulness of dietary phytochemicals has been evaluated, especially with respect to modulating deregulation of ROS-mediated signaling pathways [11]. We and others have identified potent antioxidant [6], anti-inflammatory [12] and cancer chemopreventive [13] properties of secoisolariciresinol diglucoside (SDG), a phytochemical and the main lignan in flaxseed [14,15]. Our group was the first to identify the protective properties of SDG and its synthetic version, LGM2605, in relation to the asbestos exposure of cells [16,17] and mice [18]. Specifically, LGM2605 significantly reduced asbestos-induced cytotoxicity, ROS generation, and the release of malondialdehyde (MDA) and 8-iso Prostaglandin F2α, markers of lipid peroxidation [16]. Importantly, asbestos exposure activated cellular nuclear factor-E2 related factor 2 (Nrf2), a known transcription factor that upon activation, translocates to the nucleus where it binds to the antioxidant-response element (ARE) and drives the transcription of antioxidant and detoxification genes in response to oxidative stress Nrf2. We have shown that asbestos-induced phase II antioxidant enzymes such as heme oxygenase-1 (HO-1) and NADPH: quinone oxidoreductase-1 (NQO1) were further enhanced by the action of LGM2605 [16].

The induction of antioxidant, cytoprotective enzymes under the transcriptional regulation of Nrf2 has been widely acknowledged to confer protection of normal cells from the action of chemical carcinogens [19] and from harmful ROS that are capable of damaging cellular DNA and other macromolecules such as lipids [20]. Loss of Nrf2 has been shown to enhance susceptibility to neoplasm formation from the action of chemical carcinogens [21,22], while agents that induce Nrf2 expression dramatically reduce susceptibility to carcinogenesis [23] and are thus an emerging therapeutic target. Unlike most protective agents, LGM2605 works via multiple pathways; these, as we have reported elsewhere, involve scavenging ROS/oxidants but also boosting endogenous antioxidant defenses via Nrf2 signaling as well as by blocking myeloperoxidase activity. Therefore, evaluation of LGM2605′s protective effects necessitates the investigation of its effects on several signaling molecules and cascades. In the current study, we evaluated LGM2605 in a murine model of acute asbestos-induced cytotoxicity and cell death using peritoneal macrophages from Nrf2-disrupted mice to determine the extent of the Nrf2 contribution to the protective effects of this agent. We hypothesized that in the absence of Nrf2, the protective effects of LGM2605 would be reduced.

## 2. Materials and Methods

### 2.1. Animals

The C57BL/6J mice (The Jackson Laboratory, Bar Harbor, ME, USA), which are homozygous WT for Nrf2, and Nrf2^−/−^ mice were used to derive the cells used for the current study. Nrf2-deficient C57BL/6 (RBC No. RBRC01390) mice, generated as described by Itoh et al. [24], were purchased from the RIKEN BioResource Center (Tsukuba, Japan). The genotype of each animal was confirmed by performing tail DNA extraction followed by polymerase chain reaction (PCR). PCR amplification was performed using three different primers: Nrf2-sense for both genotypes: 5′-TGGACGGGACTATTGAAGGCTG-3′; Nrf2-antisense for wild-type mice: 5′-GCCGCCTTTTCAGTAGATGGAGG-3′; Nrf2-antisense for LacZ: 5′-GCGGATTGACCGTAATGGGATAGG-3′. All animals used in the study were housed and maintained at the Children’s Hospital of Philadelphia (Philadelphia, PA, USA) following the guidelines established by the Institutional Animal Care and Use Committee (IACUC) at both the University of Pennsylvania (805791/2015) and the Children’s Hospital of Philadelphia (IAC-18-000645/2018) and the National Institutes of Health (NIH) guidelines for the care and use of laboratory animals. Mice were used at 13 weeks of age and were housed in conventional cages under standardized conditions with controlled temperature and humidity, and a 12–12-h day–night light cycle. Animals had free access to water and mouse chow.

### 2.2. Murine Peritoneal Macrophages

Murine peritoneal macrophages (MF) were harvested from the peritoneum of WT and Nrf2^−/−^ mice following elicitation using thioglycollate broth as previously described [16,17]. WT and Nrf2^−/−^ murine peritoneal macrophages were plated in 1 mL of cell culture medium (phenol-free RPMI supplemented with 1% FBS, penicillin (100 units/mL) and streptomycin (100 µg/mL), and l-Glutamine (2 mm) in a 6-well plate (2 × 10^6^ cells/well) and allowed to adhere to the bottom of the wells.

### 2.3. Crocidolite Asbestos Exposure

Elicited, peritoneal macrophages from WT and Nrf2^−/−^ mice were exposed to sterile UICC crocidolite (Na_2_O·Fe_2_O_3_·8SiO_2_·H_2_O) asbestos fibers (SPI Supplies, West Chester, PA, USA) as previously described [16,17]. Based on our previous work [16,17], elicited, peritoneal macrophages from WT and Nrf2^−/−^ mice were exposed to crocidolite asbestos fibers at a concentration of 20 µg/cm^2^.

### 2.4. Synthetic Secoisolariciresinol Diglucoside (LGM2605) Treatment

Synthesis of secoisolariciresinol diglucoside (LGM2605) has been previously described [25]. Based on our previous work [16,17], elicited, peritoneal macrophages from WT and Nrf2^−/−^ mice were treated with 50 µM and 100 µM LGM2605 4 h prior to asbestos exposure.

### 2.5. Determination of Intracellular Asbestos-Induced Reactive Oxygen Species (ROS) Generation

Levels of intracellular ROS were determined using CellROX^®^ Green Reagent (Thermo Fisher Scientific, Waltham, MA, USA). Elicited macrophages from WT and Nrf2^−/−^ mice were exposed to LGM2605 (50 µM and 100 µM) 4 h prior to asbestos challenge (20 µg/cm^2^) and cells were harvested at 24 h post asbestos exposure. Asbestos-treated and untreated cells were incubated with 5 μM CellROX^®^ Green Reagent (Thermo Fisher Scientific, Waltham, MA, USA) for 20 min at 37 °C after which cells were washed with phenol red free RPMI and imaged on a Nikon TMD fluorescence microscope (Nikon Diaphot TMD, Melville, NY, USA) equipped with a Hamamatsu ORCA-100 camera (Hamamatsu Photonics K.K., Hamamatsu City, Japan). All fluorescent cell images were acquired at the same exposure and offset settings using the MetaMorph acquisition software (Version 7.7, Molecular Devices, Downington, PA, USA). The fluorescent images of cells were processed and quantitated for CellROX^®^ Green Reagent fluorescence by the use of ImageJ software (Fiji Version, National Institutes of Health, Bethesda, MD, USA). The intensity of cells in each field was integrated to obtain the total fluorescence intensity of a particular field. Scale bar = 20 μm.

### 2.6. Determination of Asbestos-Induced Cell Death and Cytotoxicity

Asbestos-induced cell death was determined using LysoTracker^®^ Deep Red (Thermo Fisher Scientific, Waltham, MA, USA). Elicited macrophages from WT and Nrf2^−/−^ mice were exposed to LGM2605 (50 µM and 100 µM) 4 h prior to asbestos challenge (20 µg/cm^2^) and cells were harvested at 24 h post asbestos exposure. Asbestos-treated and untreated cells were incubated with 25 nM LysoTracker^®^ Deep Red (Thermo Fisher Scientific, Waltham, MA, USA) for 30 min at 37 °C after which cells were washed with PBS and fixed. The fixed cells were imaged on a Nikon TMD fluorescence microscope (Nikon Diaphot TMD, Melville, NY, USA) equipped with a Hamamatsu ORCA-100 camera (Hamamatsu Photonics K.K., Hamamatsu City, Japan). All images were acquired at the same exposure and offset settings using the MetaMorph acquisition software (Version 7.7, Molecular Devices, Downington, PA, USA). LysoTracker^®^ Deep Red is a fluorophore that selectively accumulates in acidic compartments and exhibits red fluorescence (594 nm). Red fluorescence is an indicator of increase in lysosomal vacuoles and thus represents an increase in self-digestion and apoptotic mechanisms of cell death. The fluorescent images of cells were processed and quantitated for red fluorescence by the use of ImageJ software (Fiji Version, National Institutes of Health, Bethesda, MD, USA). The intensity of cells in each field was integrated to obtain the total fluorescence intensity of a particular field. Scale bar = 20 μm.

Asbestos-induced cytotoxicity was determined by quantitatively measuring extracellular levels of lactate dehydrogenase (LDH) released into the cell culture medium at 24 h post asbestos exposure as previously described [16]. Data are reported as LDH cytotoxicity (fold change from WT control).

### 2.7. Analysis of Nitrate/Nitrite Levels in Cell Culture Medium

Levels of nitrates and nitrites, metabolites of nitric oxide, in the culture medium were determined using a nitrate/nitrite colorimetric assay kit (Cayman Chemical, Ann Arbor, MI, USA) according to the manufacturer’s protocol, as previously described [17].

### 2.8. Proinflammatory Cytokine Release

Levels of proinflammatory cytokines, IL-1β, IL-6, IL-18, and tumor necrosis factor alpha (TNFα), were determined in cell culture medium at 0 h, 8 h, and 24 h following asbestos exposure using enzyme-linked immunosorbent assays (ELISA) as previously described [17]. Samples were run undiluted in triplicate, and assays were performed according to manufacturer’s instructions. Levels of IL-1β, IL-6, IL-18, and TNFα are reported as picograms per milliliter (pg/mL) of culture medium. ELISA kits (TNFα and IL-1β) were purchased from BD biosciences (San Jose, CA, USA), MBL International (Woburn, MA, USA) (mouse IL-18 ELISA Kit), and R&D systems (Minneapolis, MN, USA) (mouse IL-6 Quantikine ELISA Kit).

### 2.9. Determination of Antioxidant Enzyme Activity and Abundance

The enzymatic activity and abundance of key antioxidant enzymes, glutathione S-transferase (GST), thioredoxin reductase (TrxR), total glutathione, and glutathione peroxidase (GPx), were evaluated in cell lysates at 0 h, 8 h, and 24 h post exposure to asbestos. Samples were run undiluted in triplicate, and assays were performed according to manufacturer’s instructions (Cayman Chemical, Ann Arbor, MI, USA).

### 2.10. RNA Isolation and Gene Expression Analysis

Total RNA isolation from WT and Nrf2^−/−^ murine peritoneal macrophages, RNA quantification, and cDNA synthesis was performed as previously described [16,17]. Individual TaqMan gene expression assays were selected for *inducible nitric oxide synthase* (*iNOS*) and relevant cytoprotective and phase II antioxidant enzymes (*thioredoxin reductase 1* (*TXNRD1*) and *glutathione S-transferase Mu 1* (*GSTM1*)). Gene expression data were normalized to ß-actin RNA housekeeping gene and calibrated to the WT control samples (WT CTL at time 0) according to the ΔΔ*C*_T_ method, as previously described [26].

### 2.11. Statistical Analysis

All data were analyzed using three-way analysis of variance (ANOVA) to test for the main effects of (a) time (8 h vs. 24 h); (b) treatment (ASB vs. ASB + LGM2605); and (c) cell type (WT vs. Nrf2^−/−^), along with the interaction between these variables, on study outcome measures. Post-tests (Tukey’s multiple comparisons tests) were conducted analyzing significant differences among treatment groups (ASB versus ASB + LGM2605) and between cell types (WT versus Nrf2^−/−^). Statistically significant differences were determined using GraphPad Prism version 6.00 for Windows, GraphPad Software, La Jolla, CA, USA, www.graphpad.com. Results are reported as mean ± the standard error of the mean (SEM). Levels of target gene mRNA are reported as the mean fold change from WT CTL at time 0 ± SEM. Statistically significant differences were determined at *p*-value of 0.05. Solid lines (―) indicate statistically significant differences between asbestos-only exposure and asbestos plus LGM2605-treatment within each respective cell type and time point. Dashed lines (---) indicate statistically significant differences between cell types (WT versus Nrf2^−/−^) within each respective treatment group and time point. Asterisks shown in figures indicate significant differences between groups (* = *p* < 0.05, ** = *p* < 0.01, *** = *p* < 0.001 and **** = *p* < 0.0001).

## 3. Results

**Figure 1:**
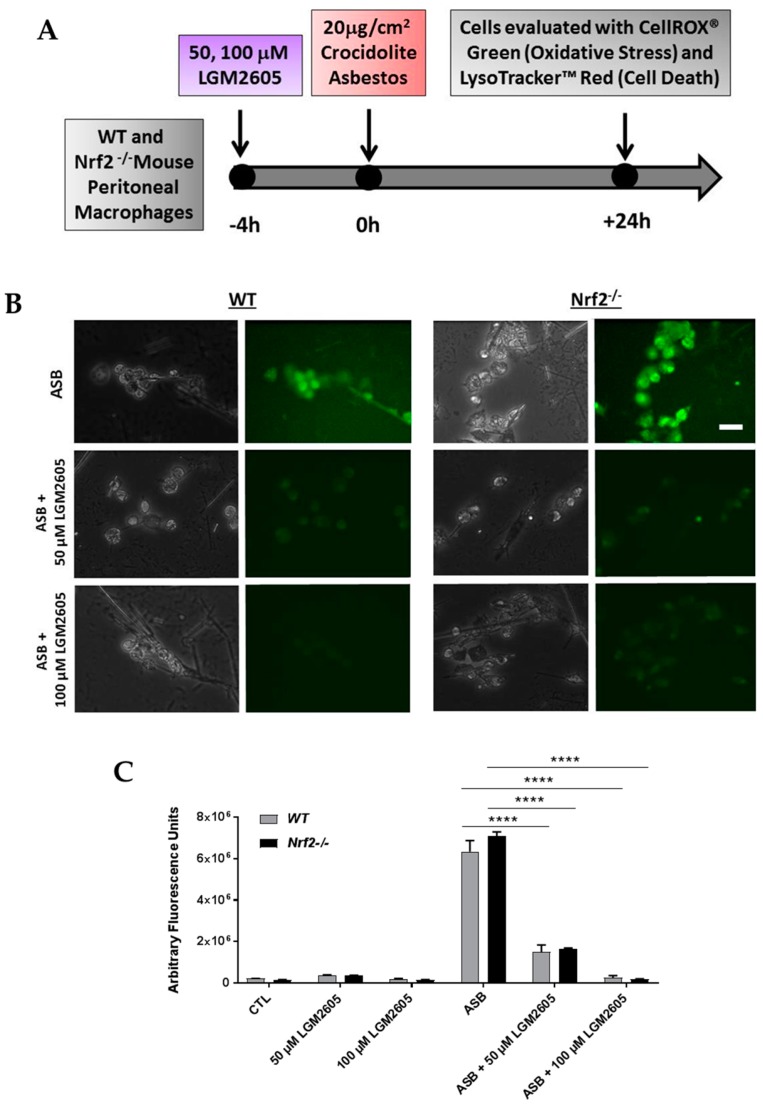
Determination of asbestos-induced oxidative stress in elicited, peritoneal macrophages from WT and Nrf2^−^^/−^ mice. Experimental plan (**A**); fluorescent images of elicited macrophages from WT and Nrf2^−^^/−^ mice exposed to LGM2605 (50 µM and 100 µM) 4 h prior to asbestos challenge (20 µg/cm^2^) and harvested at 24 h post asbestos exposure and incubated with 5 μM CellROX^®^ Green Reagent (Thermo Fisher Scientific, Waltham, MA, USA). Scale bar = 20 μm (**B**); graphed representation of the data from 3–4 fields imaged for each condition (control (CTL), 50 µM LGM2605-only, 100 µM LGM2605-only, ASB, ASB and 50 µM LGM2605, and ASB and 100 µM LGM2605) for *n* = 3 independent experiments (**C**). Data are presented as mean ± SEM. Solid lines (―) indicate statistically significant differences between asbestos-only exposure and asbestos plus LGM2605-treatment within each respective cell type. Asterisks shown in figures indicate significant differences between groups (**** = *p* < 0.0001).

To determine the contribution of Nrf2 to the cytoprotective effects of synthetic SDG (LGM2605) in preventing asbestos-induced inflammatory phenotype, inflammasome activation and oxidative cell damage and death, we utilized abdominal macrophages from Nrf2 disrupted mice (Nrf2^−/−^) as compared to WT. Elicited, abdominal murine peritoneal macrophages, a model of tissue phagocyte response to the presence of asbestos in the pleural space, were evaluated 24 h post exposure to asbestos (Figure 1A). The action of LGM2605 in mitigating the effects of asbestos exposure was evaluated by determination of ROS generation, cytotoxicity and cell death, proinflammatory cytokine secretion, antioxidant enzyme activity, and mRNA levels of Nrf2-regulated genes.

### 3.1. LGM2605 Reduces Asbestos-Induced Oxidative Stress in Elicited, Peritoneal Macrophages from WT and Nrf2^−/−^ Mice

We evaluated the effect of LGM2605 treatment on asbestos-induced intracellular ROS and oxidative stress generation using CellROX^®^ Green Reagent (Figure 1B,C). ROS generation was significantly increased at 24 h post asbestos exposure compared to control murine macrophages not exposed to asbestos. We observed a significant (*p* < 0.05), dose-dependent decrease in asbestos-induced ROS generation with LGM2605 pretreatment (76.6% decrease among WT macrophages treated with 50 µM LGM2605 and 95.9% decrease among WT macrophages treated with 100 µM LGM2605). ROS generation was equally high in WT and Nrf2^−/−^ cells, and the effect of LGM2605 on asbestos-induced ROS generation was not different across both cell types. No significant differences were observed between peritoneal macrophages from WT and Nrf2^−/−^ mice.

### 3.2. LGM2605 Decreases Asbestos-Induced Cytotoxicity and Cell Death in Elicited, Peritoneal Macrophages from WT and Nrf2^−/−^ Mice

**Figure 2:**
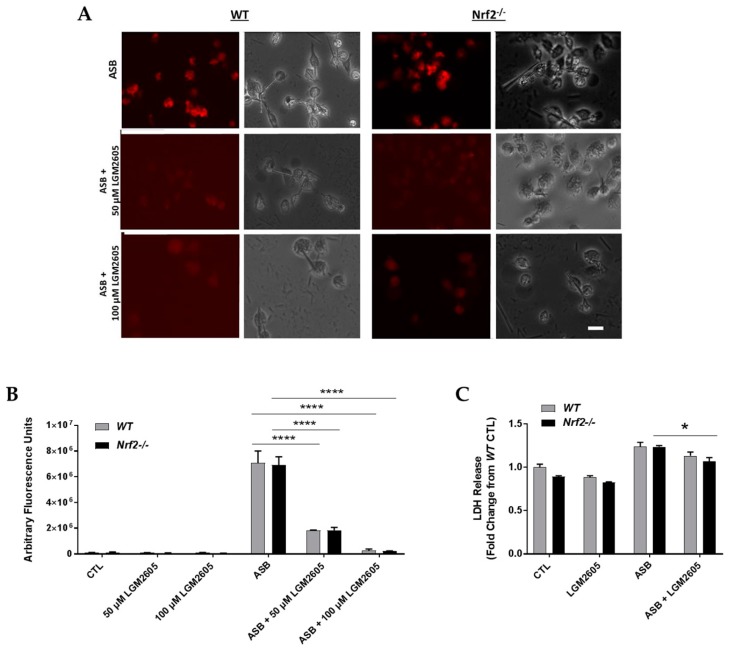
Determination of asbestos-induced cytotoxicity and cell death in elicited, peritoneal macrophages from WT and Nrf2^−^^/−^ mice. Fluorescent images of elicited macrophages from WT and Nrf2^−/−^ mice exposed to LGM2605 (50 µM and 100 µM) 4 h prior to asbestos challenge (20 µg/cm^2^) and harvested at 24 h post asbestos exposure and incubated with 25 nM LysoTracker^®^ Deep Red (Thermo Fisher Scientific, Waltham, MA, USA). Scale bar = 20 μm (**A**); graphed representation of the data from 3–4 fields imaged for each condition (control (CTL), 50 µM LGM2605-only, 100 µM LGM2605-only, ASB, ASB and 50 µM LGM2605, and ASB and 100 µM LGM2605) for *n* = 3 independent experiments (**B**); determination of LDH release at 24 h post asbestos exposure as a measure of cell death (**C**); Data are presented as mean ± SEM. Solid lines (―) indicate statistically significant differences between asbestos-only exposure and asbestos plus LGM2605-treatment within each respective cell type and time point. Asterisks shown in figures indicate significant differences between groups (* = *p* < 0.05 and **** = *p* < 0.0001).

Asbestos-induced cell death and cytotoxicity was determined at 24 h post asbestos exposure. Figure 2A,B depict fluorescent labelling and quantification of live cells using LysoTracker^®^ Deep Red. As seen in Figure 2A, exposure to 20 µg/cm^2^ of crocidolite asbestos induces significant cell death 24 h post exposure. The level of asbestos-induced cell death was not significantly different between WT and Nrf2^−/−^ macrophages (Figure 2A). Pretreatment of cells with 50 µM and 100 µM LGM2605, significantly (*p* < 0.05) prevented cell death and cytotoxicity in cells from WT and Nrf2^−/−^ mice (74.3% and 96.3% decrease among WT macrophages treated with 50 µM and 100 µM LGM2605, respectively, and 73.4% and 96.9% decrease among Nrf2^−/−^ macrophages treated with 50 µM and 100 µM LGM2605, respectively). Protection by LGM2605 was dose-dependent and independent from a functional Nrf2.

Levels of lactate dehydrogenase (LDH), an enzyme released into the cell culture medium from damaged cells, were determined at 24 h post asbestos exposure for WT and Nrf2^−/−^ macrophages (Figure 2C). Levels of LDH release remained low following exposure to LGM2605-alone. Asbestos exposure led to a significant (*p* < 0.05) increase in LDH released into the cell culture medium for both WT and Nrf2^−/−^ macrophages. Among asbestos exposed Nrf2^−/−^ macrophages, pretreatment with 50 µM LGM2605 led to a significant (*p* = 0.0081) decrease in LDH compared to asbestos-only exposure.

### 3.3. LGM2605 Reduces Asbestos-Induced Nitrosative Stress in Elicited Macrophages from WT and Nrf2^−/−^ Mice

**Figure 3:**
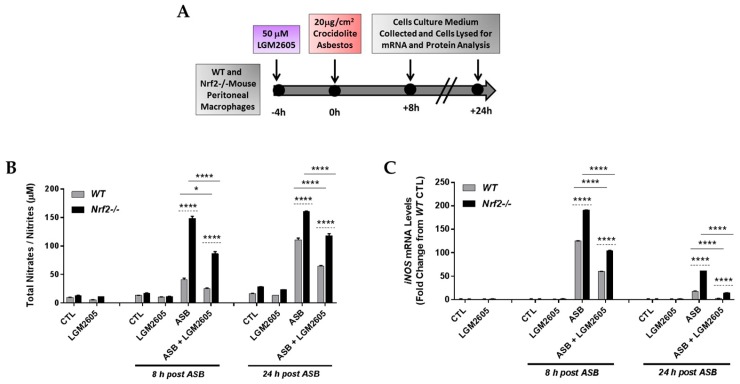
Evaluation of LGM2605 inhibition of nitrosative stress in elicited, peritoneal macrophages exposed to asbestos. Elicited macrophages from WT and Nrf2^−/−^ mice were exposed to LGM2605 (50 µM) 4 h prior to asbestos challenge (20 µg/cm^2^) and cells were harvested and cell culture medium collected at 8 h and 24 h post asbestos exposure (**A**); total nitrates/nitrites in cell culture medium (**B**) and cellular *iNOS* mRNA levels (**C**) were evaluated at 8 and 24 h post exposure to asbestos. Samples were run undiluted in triplicate and data are presented as mean ± SEM. Solid lines (―) indicate statistically significant differences between asbestos-only exposure and asbestos plus LGM2605-treatment within each respective cell type and time point. Dashed lines (---) indicate statistically significant differences between cell types (WT versus Nrf2^−/−^) within each respective treatment group and time point. Asterisks shown in figures indicate significant differences between groups (* = *p* < 0.05 and **** = *p* < 0.0001).

LGM2605 treatment (50 µM) was initiated 4 h prior to exposure to crocidolite asbestos fibers (20 µg/cm^2^) and cell culture medium and macrophages were collected at 0 h, 8 h, and 24 h post asbestos exposure (Figure 3A). Levels of total nitrates/nitrites were determined in the cell culture medium following asbestos exposure (Figure 3B). Among WT macrophages, asbestos exposure led to increased levels of total nitrates/nitrites relative to unexposed macrophages at 24 h post asbestos exposure (10.15 µM ± 0.10 µM compared to 110.70 µM ± 4.90 µM). Nitrosative stress was much more profound in asbestos-exposed Nrf2^−/−^ macrophages than in WT macrophages suggesting enhanced sensitivity (41.47 µM ± 2.08 µM compared to 148.86 µM ± 3.44 µM at 8 h post asbestos and 110.70 µM ± 4.90 µM compared to 160.72 µM ± 0.86 µM at 24 h post asbestos). In fact, sensitivity was higher by 3.59-fold and 1.45-fold over control at 8 and 24 h, respectively (Figure 3A,B). Interestingly, LGM2605 significantly (*p* < 0.05) reduced nitrosative stress in both WT and Nrf2^−/−^ macrophages.

Levels of *inducible nitric oxide synthase* (*iNOS*) mRNA were significantly elevated at 8 h post asbestos exposure (Figure 3C). Similar to the levels of total nitrates/nitrites, which were significantly higher in Nrf2^−/−^ macrophages than in WT macrophages, mRNA levels of *iNOS* were also significantly (*p* < 0.0001) higher in Nrf2^−/−^ macrophages. Treatment with LGM2605 4 h prior to asbestos exposure significantly reduced mRNA levels of *iNOS* in both WT and Nrf2^−/−^ macrophages.

### 3.4. The Synthetic SDG (LGM2605) Reduces Asbestos-Induced Inflammasome Activation in Elicited Macrophages from WT, but Not Nrf2^−/−^, Mice

**Figure 4:**
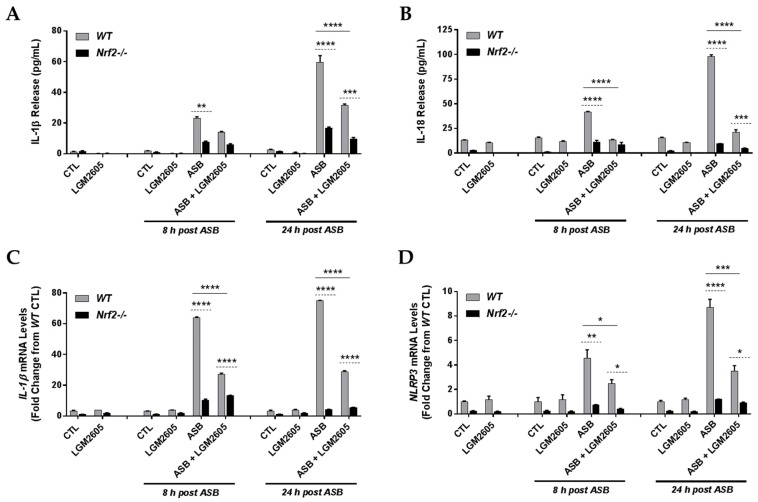

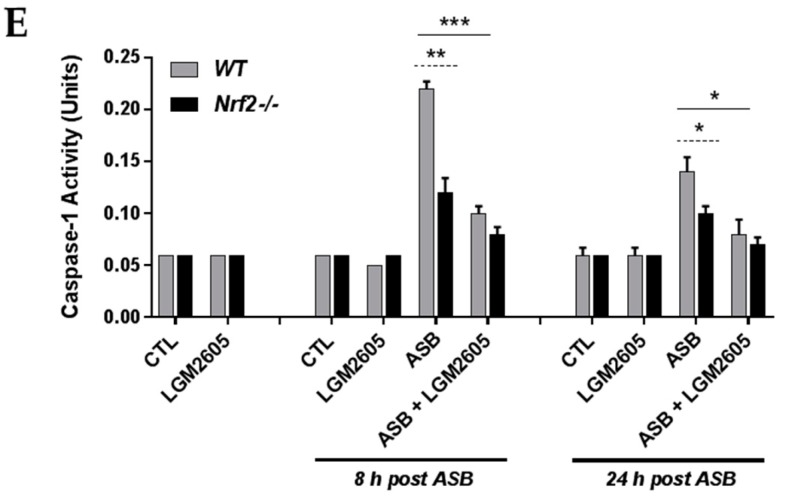
Evaluation of LGM2605 inhibition of proinflammatory cytokine release and inflammasome activation in elicited, peritoneal macrophages exposed to asbestos. Elicited macrophages from WT and Nrf2^−/−^ mice were exposed to LGM2605 (50 µM) 4 h prior to asbestos challenge (20 µg/cm^2^) and cells were harvested and cell culture medium collected at 8 h and 24 h post asbestos exposure. Inflammasome-relevant cytokine release, IL-1β (**A**) and Il-18 (**B**); cellular mRNA levels of *IL-1β* (**C**) and *NLRP3* (**D**); and caspase-1 activity (**E**) were evaluated at 8 and 24 h post exposure to asbestos. Samples were run undiluted in triplicate and data are presented as mean ± SEM. Solid lines (―) indicate statistically significant differences between asbestos-only exposure and asbestos plus LGM2605-treatment within each respective cell type and time point. Dashed lines (---) indicate statistically significant differences between cell types (WT versus Nrf2^−/−^) within each respective treatment group and time point. Asterisks shown in figures indicate significant differences between groups (* = *p* < 0.05, ** = *p* < 0.01, *** = *p* < 0.001 and **** = *p* < 0.0001).

Activation of the NLRP3 inflammasome by asbestos was shown by many investigators including our team [17]. Strategies to inhibit NLRP3 are being explored as a means to mitigate the inflammatory response to asbestos that has been linked to malignancy. NLRP3 priming, caspase-1 activity, and levels of inflammatory cytokines generated by NLRP3 inflammasome activation were evaluated in both WT and Nrf2^−/−^ macrophages (Figure 4). We observed a time-dependent increase in both IL-1β (Figure 4A) and IL-18 (Figure 4B) at 8 h and 24 h post asbestos exposure among WT macrophages (from 1.39 pg/mL ± 0.15 pg/mL at baseline to 23.08 pg/mL ± 1.01 pg/mL at 8 h post asbestos to 59.55 pg/mL ± 4.37 pg/mL at 24 h post asbestos for IL-1β and from 13.07 pg/mL ± 0.27 pg/mL at baseline to 41.50 pg/mL ± 0.63 pg/mL at 8 h post asbestos to 97.67 pg/mL ± 2.00 pg/mL at 24 h post asbestos for IL-18). Compared to WT macrophages, levels of IL-1β and IL-18 following asbestos exposure were significantly less for Nrf2^−/−^ macrophages. Pretreatment with LGM2605 significantly (*p* < 0.0001) reduced levels of IL-1β and IL-18 at 24 h post asbestos among WT macrophages by 46.7% and 78.2%, respectively.

Although not statistically significantly different, Nrf2^−/−^ macrophages treated with LGM2605 had reduced levels of IL-1ß release at 8 h and 24 h post asbestos exposure (7.78 pg/mL ± 0.60 pg/mL for asbestos-only exposure compared to 5.94 pg/mL ± 0.87 pg/mL for asbestos-exposed and LGM2605-treated macrophages at 8 h post asbestos and 16.77 pg/mL ± 0.92 pg/mL for asbestos-only exposure compared to 9.55 pg/mL ± 1.40 pg/mL for asbestos-exposed and LGM2605-treated macrophages at 24 h post asbestos). Similar findings were observed for IL-18 release, a cytokine whose activation is mediated by active caspase-1. Although not statistically significantly different, Nrf2^−/−^ macrophages treated with LGM2605 had reduced levels of IL-18 release at 8 h and 24 h post asbestos exposure (11.37 pg/mL ± 2.12 pg/mL for asbestos-only exposure compared to 8.73 pg/mL ± 2.83 pg/mL for asbestos-exposed and LGM2605-treated macrophages at 8 h post asbestos and 9.47 pg/mL ± 0.28 pg/mL for asbestos-only exposure compared to 4.67 pg/mL ± 0.66 pg/mL for asbestos-exposed and LGM2605-treated macrophages at 24 h post asbestos).

Additionally, mRNA levels of *IL-1β* (Figure 4C) and *NLRP3* (Figure 4D) were significantly elevated following asbestos exposure, with mRNA levels being significantly higher among WT macrophages relative to Nrf2^−/−^ macrophages. LGM2605 significantly reduced mRNA levels of both *IL-1β* and *NLRP3* among WT macrophages, but not among Nrf2^−/−^ macrophages. Caspase-1 activity was elevated at 8 h and 24 h following asbestos exposure, with increased activity observed among WT macrophages compared to Nrf2^−/−^ macrophages. Among WT macrophages, LGM2605 pretreatment significantly reduced caspase-1 activity at both 8 h and 24 h post asbestos (Figure 4E).

### 3.5. The Synthetic SDG (LGM2605) Reduces Asbestos-Induced Inflammatory Cytokine Release in Elicited Macrophages from WT and Nrf2^−/−^ Mice

**Figure 5:**
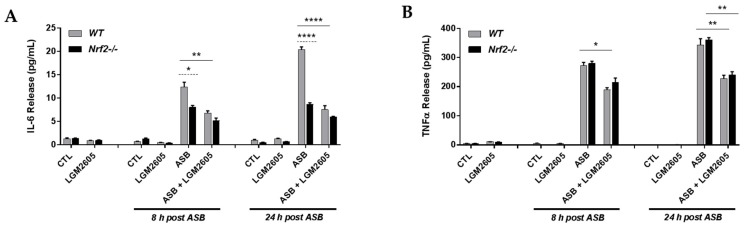
Evaluation of LGM2605 inhibition of inflammatory cytokine release in elicited, peritoneal macrophages exposed to asbestos. Elicited macrophages from WT and Nrf2^−/−^ mice were exposed to LGM2605 (50 µM) 4 h prior to asbestos challenge (20 µg/cm^2^) and cells were harvested and cell culture medium collected at 8 h and 24 h post asbestos exposure. Inflammatory cytokine release, IL-6 (**A**) and TNFα (**B**), were evaluated at 8 and 24 h post exposure to asbestos. Samples were run undiluted in triplicate and data are presented as mean ± SEM. Solid lines (―) indicate statistically significant differences between asbestos-only exposure and asbestos plus LGM2605-treatment within each respective cell type and time point. Dashed lines (---) indicate statistically significant differences between cell types (WT versus Nrf2^−/−^) within each respective treatment group and time point. Asterisks shown in figures indicate significant differences between groups (* = *p* < 0.05, ** = *p* < 0.01, and **** = *p* < 0.0001).

Having observed an increase in the levels of proinflammatory cytokines produced by NLRP3 inflammasome activation, such as IL-1β and IL-18, following asbestos exposure, we further evaluated the cell culture medium for levels of proinflammatory cytokines, such as IL-6 (Figure 5A) and TNFα (Figure 5B). As shown in Figure 5, asbestos exposure led to an increase in both IL-6 and TNFα at 8 h and 24 h post exposure among WT macrophages (from 1.29 pg/mL ± 0.21 pg/mL at baseline to 12.41 pg/mL ± 1.00 pg/mL at 8 h post asbestos to 20.37 pg/mL ± 0.58 pg/mL at 24 h post asbestos for IL-6 and from 4.78 pg/mL ± 1.11 pg/mL at baseline to 273.43 pg/mL ± 9.96 pg/mL at 8 h post asbestos to 343.26 pg/mL ± 21.62 pg/mL at 24 h post asbestos for TNFα). Among WT macrophages, LGM2605 treatment significantly reduced levels of both IL-6 and TNFα at 8 h and 24 h post asbestos exposure by 45.0% and 62.9%, respectively, for IL-6 and by 30.6% and 33.4%, respectively, for TNFα. Significant differences in IL-6 were observed between WT and Nrf2^−/−^ macrophages, with Nrf2^−/−^ macrophages displaying reduced levels of IL-6 (Figure 5A). Alternatively, no significant differences in TNFα levels were observed between WT and Nrf2^−/−^ macrophages (Figure 5B).

### 3.6. Exposure to Crocidolite Asbestos Fibers Induces Phase II Antioxidant Enzyme Expression in Elicited Macrophages from WT and Nrf2^−/−^ Mice

**Figure 6:**
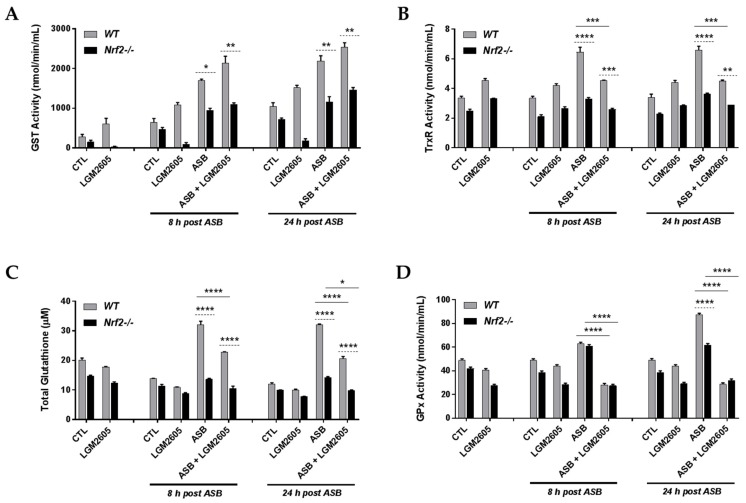
Asbestos exposure induces the activity and abundance of antioxidant enzymes. Elicited macrophages from WT and Nrf2^−/−^ mice were exposed to LGM2605 (50 µM) 4 h prior to asbestos challenge (20 µg/cm^2^) and cells were harvested and cell culture medium collected at 8 h and 24 h post asbestos exposure. The enzymatic activity and abundance of key antioxidant enzymes, glutathione S-transferase (GST) (**A**); thioredoxin reductase (TrxR) (**B**); total glutathione (**C**); and glutathione peroxidase (GPx) (**D**), were evaluated at 8 and 24 h post exposure to asbestos. Samples were run undiluted in triplicate and data are presented as mean ± SEM. Solid lines (―) indicate statistically significant differences between asbestos-only exposure and asbestos plus LGM2605-treatment within each respective cell type and time point. Dashed lines (---) indicate statistically significant differences between cell types (WT versus Nrf2^−/−^) within each respective treatment group and time point. Asterisks shown in figures indicate significant differences between groups (* = *p* < 0.05, ** = *p* < 0.01, *** = *p* < 0.001 and **** = *p* < 0.0001).

To further investigate the differential effects of LGM2605 pretreatment on asbestos-exposed WT and Nrf2^−/−^ macrophages, we evaluated the enzymatic activity and abundance of key Nrf2-regulated antioxidant enzymes, glutathione S-transferase (GST), thioredoxin reductase (TrxR), total glutathione, and glutathione peroxidase (GPx). As seen in Figure 6, asbestos-only exposure led to a significant (*p* < 0.05) increase in GST activity (Figure 6A), TrxR activity (Figure 6B), total glutathione (Figure 6C), and GPx activity (Figure 6D) among WT macrophages. LGM2605 pretreatment led to a significant (*p* < 0.001) decrease in TrxR activity, total glutathione, and GPx activity among WT macrophages. Importantly, the observed response (increases in antioxidant enzyme activity and abundance) with asbestos exposure among WT macrophages was significantly attenuated among Nrf2^−/−^ macrophages. 

Indeed, in the absence of asbestos exposure, pretreatment with LGM2605-alone led to significantly reduced GST activity when compared to CTL (no asbestos exposure and not treated with LGM2605) in Nrf2^−/−^ macrophages. Specifically, LGM2605 pretreatment significantly (*p* = 0.045) reduced GST activity from CTL (472.53 nmol/min/mL ± 62.48 nmol/min/mL for CTL compared to 85.48 nmol/min/mL ± 69.27 nmol/min/mL for LGM2605-only at 8 h post asbestos exposure). While GST activity is reduced among Nrf2^−/−^ macrophages as compared to WT macrophages, pretreatment with LGM2605, led to a further reduction in GST activity among Nrf2^−/−^ macrophages and not WT macrophages. As LGM2605 has potent free radical scavenging capabilities, the presence of the drug, administered 4 h prior to asbestos exposure, may further downregulate the need for endogenous cellular antioxidant enzymes, such as GST. This is further supported by the reduced activity of other endogenous cellular antioxidant enzymes, such as GPx (Figure 6D), with LGM2605-only treatment as compared to CTL among Nrf2^−/−^ macrophages.

### 3.7. LGM2605 Enhances Asbestos-Induced Gene Expression of Phase II Enzymes in Elicited Macrophages from WT and Nrf2^−/−^ Mice

**Figure 7:**
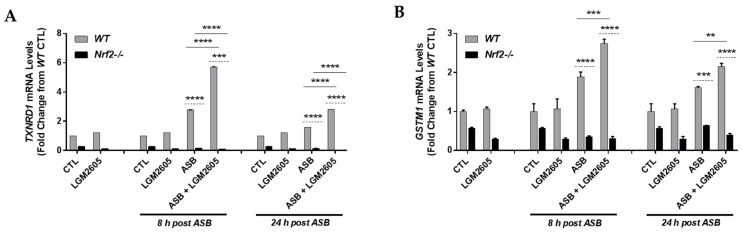
Asbestos exposure induces the expression of phase II antioxidant enzymes. Elicited macrophages from WT and Nrf2^−/−^ mice were exposed to LGM2605 (50 µM) 4 h prior to asbestos challenge (20 µg/cm^2^) and cells were harvested and cell culture medium collected at 8 h and 24 h post asbestos exposure. The mRNA levels of phase II antioxidant enzymes, *thioredoxin reductase 1* (*TXNRD1*) (**A**) and *glutathione S-transferase Mu 1* (*GSTM1*) (**B**), were evaluated at 8 h and 24 h post exposure to asbestos. Samples were run undiluted in triplicate and data are presented as mean ± SEM. Solid lines (―) indicate statistically significant differences between asbestos-only exposure and asbestos plus LGM2605-treatment within each respective cell type and time point. Dashed lines (---) indicate statistically significant differences between cell types (WT versus Nrf2^−/−^) within each respective treatment group and time point. Asterisks shown in figures indicate significant differences between groups (** = *p* < 0.01, *** = *p* < 0.001 and **** = *p* < 0.0001).

After evaluating the effects of asbestos exposure and LGM2605 treatment on antioxidant enzyme abundance and activity for both WT and Nrf2^−/−^ macrophages, we proceeded to determine the mRNA levels of Nrf2-regulated antioxidant enzymes, *thioredoxin reductase 1* (*TXNRD1*) (Figure 7A) and *glutathione S-transferase Mu 1* (*GSTM1*) (Figure 7B). Levels of *TXNRD1* and *GSTM1* mRNA were significantly (*p* < 0.05) elevated at 8 h and 24 h post asbestos exposure among WT macrophages. Furthermore, pretreatment with LGM2605 significantly (*p* < 0.01) boosted mRNA levels of *TXNRD1* and *GSTM1* at 8 h and 24 h post asbestos by 106.1% and 76.4%, respectively, for *TXNRD1* and by 45.2% and 33.8%, respectively, for *GSTM1*. Levels of *TXNRD1* and *GSTM1* mRNA were significantly (*p* < 0.001) reduced at 8 h and 24 h post asbestos for Nrf2^−/−^ macrophages compared to WT macrophages. In fact, asbestos-induced increases in the mRNA levels of *TXNRD1* and *GSTM1* were not observed among Nrf2^−/−^ macrophages. Indeed, pretreatment with the drug alone did not significantly increase mRNA expression of *TXNRD1* and *GSTM1*, however, the interaction of asbestos exposure and LGM2605 pretreatment led to significantly increased levels of both *TXNRD1* and *GSTM1*, above the individual effects of asbestos-only exposure and LGM2605-only treatment. In fact, the observed induction of *TXNRD1* and *GSTM1* suggests that the interaction of asbestos exposure and LGM2605 pretreatment is multiplicative, rather than additive.

## 4. Discussion

The important role of the Nrf2 signaling pathway in regulating oxidative stress and inflammation has long been identified and confirmed in a number of biological models of cardiovascular disease, diabetes or neurodegenerative diseases [27,28,29], as well as cancer [30]. Activation of Nrf2 is linked to the increased expression of genes regulating antioxidant and cytoprotective proteins such as phase I and II electrophile detoxification enzymes. Nrf2 is also involved in regulating the transport of toxic solutes, in the metabolism of free radicals, inhibition of inflammation, glutathione homeostasis, proteasome function, the recognition of DNA damage and the expression of diverse cell factors regulating differentiation and growth inhibition [31]. 

We have identified that biphenolic SDG from flaxseed [14,15,32,33] which, similar to other known bioactive phytochemicals such as sulforaphane and curcumin, acts in part via activation of the Nrf2/ARE pathway to protect biomolecules and tissues from diverse stressors [30]. SDG has also been shown to inhibit activation of the NLRP3 inflammasome [16,17] which was shown to be important in asbestos-induced inflammation [34,35]. The inflammasomes are large protein complexes that upon assembly elicit an inflammatory response to internal and external stress signals. Studies have shown that expression of Nrf2 is needed for NLRP3 activation [36] although this remains controversial. Our study aimed to determine whether the anti-inflammatory properties of SDG in asbestos-induced inflammation which were shown to be linked to inflammasome inhibition, are also dependent on a functional Nrf2. Our current findings using macrophages from Nrf2^−/−^ mice showed that increased oxidative stress and cell death/cytotoxicity induced by asbestos was of similar magnitude in WT as in Nrf2^−/−^ cells, while nitrosative stress was significantly higher in the absence of Nrf2. Importantly, LGM2605 significantly reduced the asbestos response in WT and Nrf2^−/−^ cells, showing that it protects in an Nrf2-independent mechanism. NLRP3 inflammasome activation by asbestos was significantly attenuated in Nrf2^−/−^ cells as compared to WT, however LGM2605 did not reduce the activation to background levels, an indication that it requires a functional Nrf2.

Oxidative stress and inflammation have been shown be linked in several pathologies [37,38]. Indeed, ROS and oxidative stress have been reported to upregulate inflammatory moieties, one of them being the inflammasomes of the nucleotide-binding oligomerization domain (NOD)-like receptor protein 3 family [38]. The NLRP3 pathway is one of the major inflammatory response pathways. Upon activation, the NLRP3 recruits the adaptor protein apoptosis-associated speck-like protein containing a C-terminal caspase recruitment domain (ASC); this complex activates caspase-1, leading to the processing of pro-IL-1β to form the cytotoxic IL-1β [37,39]. Among the cellular defense systems against oxidative stress and inflammation is the Nrf2 system that can, via its induction of antioxidant cascades, repress inflammation and injury. In some models of injury, the Nrf2 system has been found to engage in crosstalk with the NLRP3 inflammasome [40,41]. Our earlier studies show the pluripotency of LGM2605 via various pathways; primary among them being the Nrf2 induced expression of antioxidants. Here we report on LGM2605’s ability to decrease the expression of the NLRP3 inflammasome, a critical component of the cellular inflammation machinery. Studies elsewhere on asbestos-induced injury have shown that NLRP3 inflammasome activation in macrophages is directly linked to the release of critical cytokines associated with lung injury and pleural diseases [8]. The abrogation of the NLRP3 inflammasome by LGM2605 probably occurs via the involvement of ROS and Nrf2 pathways, but our aim was to show that it represents the protective action of this agent against asbestos-induced inflammatory lung diseases.

**Figure 8:**
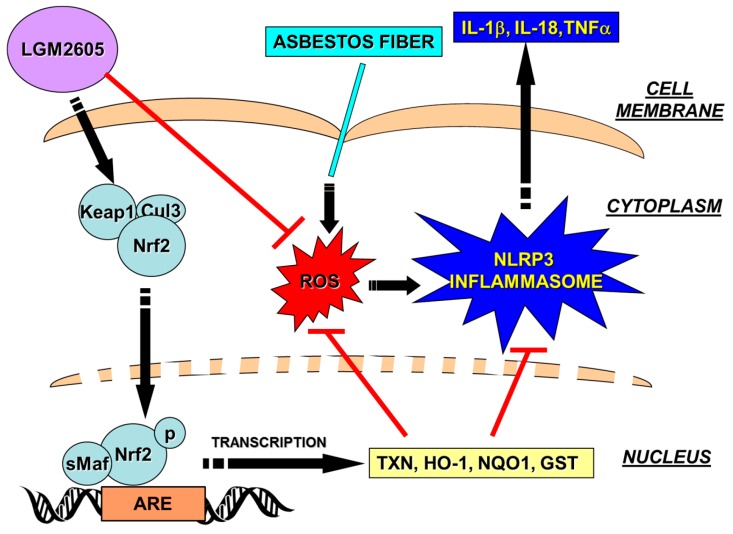
Schematic representation of LGM2605 inhibition of asbestos-induced NLRP3 inflammasome activation by blocking asbestos-induced reactive oxygen species (ROS) generation and activating the Nrf2-ARE pathway. Arrow-headed lines indicate activation and bar-headed lines indicate inhibition. ARE, antioxidant response element; Cul3, cullin-3; GST, glutathione S-transferase; HO-1, heme oxygenase-1; IL-1β, interleukin-1β; IL-18, interleukin-18; Keap1, kelch-like ECH-associated protein 1; LGM2605, synthetic SDG; NQO1, NADPH: quinone oxidoreductase-1; NLRP3, NACHT, LRR and PYD domains-containing protein 3; Nrf2, nuclear factor (erythroid-derived 2)-like 2; ROS, reactive oxygen species; sMaf, small Maf (musculoaponeurotic fibrosarcoma); TNFα, tumor necrosis factor alpha; Trx, thioredoxin.

Lack of Nrf2 has been reported to cause reduced activation of the NLRP3 and AIM2 inflammasome [39]. In our asbestos-induced oxidative stress model, activation of the NLRP3 inflammasome, as monitored by NLRP3 expression (Figure 4D), IL-1β and IL-18 release (Figure 4A,B) and by caspase-1 expression (Figure 4E), was significantly attenuated in Nrf2^−/−^ cells. LGM2605 pretreatment also inhibited the NLRP3 pathway; however, the inhibitory effect of LGM2605 was unaffected in Nrf2^−/−^ cells indicating that LGM2605 protection against NLRP3 activation was primarily via the Nrf2 pathway (Figure 8). Elsewhere too, several flavonoids and phytochemicals that have shown protection against inflammation have been found to do so via an Nrf2-dependent pathway. For instance, the flavonoid isoliquiritigenin, protected oxidant and endotoxin induced NLRP3 in lungs via the Nrf2 pathway [42] as did epigallocatechin-3-gallate (EGCG) the major bioactive polyphenol in green tea [43]. However certain natural antioxidants and anti-inflammatory compounds, such as sulforaphane, inhibit the inflammasome in an Nrf2-independent manner [44].

Asbestos-induced nitrosative stress has been identified in preclinical studies as well as in patients with asbestos exposure-related diseases [45,46]. This is further exacerbated in cells lacking a functional Nrf2, such as the ones used in our current study. Nrf2 knockdown is known to enhance the activation of the nuclear factor-κB (NF-κB) pathway in diverse cell types, which has been linked to increased iNOS expression and nitrosative stress, as well as to enhanced oxidative stress and inflammation [47]. Indeed, we see increased *iNOS* expression and nitrosative stress in asbestos-treated WT and Nrf2^−/−^ macrophages (Figure 3). While inflammasome-relevant cytokines are not induced in knockout animals (Figure 4), inflammatory cytokines such as IL-6 and TNFα are being induced (Figure 5). Indeed, TNFα can be induced via pathways independently of a functional Nrf2. Specifically, NF-κB can be activated to induce TNFα expression. On the other hand, IL-6 induction is closely linked to inflammasome activation, which may explain why it is moderately blunted in the absence of Nrf2. LGM2605, similar to other Nrf2-acting botanicals such as sulphoraphane, exerted its antioxidative and anti-inflammatory effects, in part, via upregulation of Nrf2 targets in WT macrophages.

Interestingly, Fattman et al. (2006) noted that lungs from endothelial cell superoxide dismutase (SOD)-null mice exposed to asbestos showed greater nitrosative damage, as assessed by nitrotyrosine content compared to those of their wild-type counterparts supporting the hypothesis that depletion of this Nrf2-regulated antioxidant enzyme from the lung increases oxidative stress and injury in response to asbestos [48]. Similarly, in our study, Nrf2^−/−^ cells had a higher nitrosative stress than the WT cells when exposed to asbestos as evidenced by the high nitrite/nitrate index and *inducible nitric oxide synthase* (*iNOS*) expression. LGM2605 significantly reduced nitrosative stress in WT and Nrf2^−/−^ cells, an indication that it does so, independently from a functional Nrf2. Indeed, SDG and LGM2605 were shown to work via inhibition of NF-κB [17], a redox-sensitive pro-inflammatory transcription factor which regulates numerous pathways implicated in inflammation and cancer [49]. Genes such as *iNOS* contain in their promoter region NF-κB binding sites [50], therefore, the action of LGM2605 to reduce nitrosative stress independently of a functional Nrf2 may be explained in part by the inhibition of NF-κB. This mechanism of action is shared by many phenolic compounds such as the flavonoids [51].

In regard to the differential expression between mRNA levels and enzyme activity, the specific point in time of sampling and analysis are crucial when evaluating the gene expression and enzymatic activity of Nrf2-ARE controlled targets. LGM2605-alone did not significantly induce the mRNA expression of *TXNRD1* and *GSTM1* at 0 h, 8 h, or 24 h post asbestos exposure. Of note, while mRNA levels of *TXNRD1* and *GSTM1* were elevated at 8 h post asbestos exposure among WT macrophages treated with LGM2605 and exposed to asbestos, enzymatic activity of TrxR and GPx follow differential expression patterns. For example, regarding Nrf2-ARE targets, differential expression between gene transcripts and protein levels was observed when Davidson et al. evaluated both the mRNA levels and protein levels of hepatic GST Ya from rats treated with oltipraz [52]. Similarly, Thimmulappa et al. observed differential expression between mRNA transcripts and enzyme activities of G6PDH and malic enzyme in small intestine from wild-type and Nrf2^−/−^ mice treated with sulforaphane [53]. Thus, the apparent disparity between the action of the drug on enzyme activity and gene expression may be due to differential regulation by the enzyme. Furthermore, under conditions of oxidative stress, the disparity between enzyme activity and gene expression has been shown earlier [54]. Specifically, exposure to asbestos fibers leads to high oxidative stress and although LGM2605 treatment increases antioxidant protein expression, such protein (synthesized in an oxidative stress environment) is probably in an inactive state. Therefore, it is likely that the excess protein detected is an inactive version. Such increase in protein expression with a concomitant decrease in activity is a well-documented phenomenon in several models of disease that are associated with severe oxidative injury [54,55,56].

A great number of occupations expose workers daily to hazardous environments that contain asbestos or asbestos-containing materials [57]. Such occupations include plumbers, electricians, construction workers, insulators and, in general, those that work in old buildings with asbestos. Of additional importance, as a risk factor for mesothelioma development, is also para-occupational exposure of members of the worker’s household, which could handle contaminated clothing [58]. On the basis of such observations, preventative measures need to be taken during work. Our studies, where LGM2605 was evaluated as a preventive agent, aimed to provide insight to the usefulness of this agent in this and other similar exposures. Our findings complement previous work in asbestos-exposed mice whereby SDG-rich diets given preventively mitigated asbestos-induced inflammation and damage [18].

LGM2605 and natural SDG were shown to be protective via mechanisms that do not involve the Nrf2 pathway. It has been known for several decades that SDG is a potent free radical scavenger, a quality we confirmed in its synthetic version, LGM2605 [25,33]. We have recently shown that LGM2605 is also a potent scavenger of active chlorine species (ACS), generated by radiation of physiological solutions [32]. We extended those initial findings to now show that importantly, LGM2605 inhibits myeloperoxidase (MPO), the key enzyme in inflammatory cells such as macrophages and neutrophils that generates hypochlorous acid (HOCl) during inflammation and infection [59]. LGM2605, by inhibiting MPO, decreases generation of HOCl that causes chlorination and oxidation of nucleobases and proteins, i.e., damages macromolecules vital for cell survival. In addition, we have shown that LGM2605 scavenges HOCl as well as •OH free radicals that produce ACS by reacting with chloride ions. The action of LGM2605 as an MPO inhibitor and ACS/ROS scavenger may explain the protective effects of this agent in asbestos-induced cytotoxicity, independently from a functional Nrf2.

## 5. Conclusions

In conclusion, the current study identified Nrf2-dependent and independent protective action of LGM2605 in asbestos-induced cytotoxicity and cell death. This further confirms the usefulness of this agent in mitigating inflammation and oxidative tissue damage from asbestos exposure that are linked to malignancy.

## Abbreviations

ARE	antioxidant response element
ASC	apoptosis-associated speck-like protein containing a C-terminal caspase recruitment domain
CTL	control
ED	enterodiol
EL	enterolactone
ELISA	enzyme-linked immunosorbent assay
FLC	flaxseed lignan component
*GSTM1*	*glutathione S-transferase Mu 1*
GPx	glutathione peroxidase
HO-1	heme oxygenase-1
IL-18	interleukin-18
IL-1β	interleukin-1β
IL-6	interleukin-6
IACUC	Institutional Animal Care and Use Committee
iNOS	inducible nitric oxide synthase
IP	intraperitoneal
KEAP1	kelch-like ECH-associated protein 1
LDH	lactate dehydrogenase
MF	macrophage
MDA	malondialdehyde
MM	malignant mesothelioma
NF-κB	nuclear factor-κB
NLRP3	nod-like receptor family pyrin domain containing 3
NOD	nucleotide-binding oligomerization domain
Nrf2	nuclear factor (erythroid-derived 2)-like 2
Nqo1	NADPH: quinone oxidoreductase-1
PBS	phosphate-buffered saline
PL	peritoneal lavage
PLF	peritoneal lavage fluid
qPCR	quantitative polymerase chain reaction
RNS	reactive nitrogen species
ROS	reactive oxygen species
SDG	secoisolariciresinol diglucoside
TNFα	tumor necrosis factor alpha
TrxR	thioredoxin reductase
WBC	white blood cell
